# Analysis of factors influencing hospital-acquired infection in postoperative patients with intracranial aneurysm

**DOI:** 10.1186/s12883-019-1565-2

**Published:** 2019-12-20

**Authors:** Jun Wang, Yuanyuan Ji, Lidan Jiang, Xia Zhao, Shaochen Guan, Piao Yang, Jie Yu, Yunyun Liu, Hongqi Zhang

**Affiliations:** 1 Department of Neurosurgery, Xuanwu Hospital, Capital Medical University, China International Neuroscience Institute (China-INI), 45 Changchun Street, Beijing, 100053 China; 2 Hospital Infection Management Division, Xuanwu Hospital, Capital Medical University, Beijing, China; 3 Centre for Evidence-Based Medicine, Xuanwu Hospital, Capital Medical University, Beijing, China

**Keywords:** Intracranial aneurysm, Hospital-acquired infection, Risk factor, Case-control study

## Abstract

**Background:**

Hospital-acquired infection (HAI) is a serious complication of neurosurgery. In recent years, the medical body has paid increasing attention to this issue.

**Aim:**

We investigated the status of HAIs in patients who had undergone surgery for intracranial aneurysms and analysed their risk factors.

**Methods:**

A retrospective analysis was carried out on the medical records of 542 patients with intracranial aneurysms after they were admitted for neurosurgery at Xuanwu Hospital of Capital Medical University between January and December 2016. Cases studied were divided into an infection group and a control group. Logistic regression analysis of the data was carried out.

**Findings:**

Of the 542 patients with intracranial aneurysms who underwent surgery, 77 HAIs occurred in 64 patients, with an infection prevalence of 11.8% and prevalence of infection cases of 14.2%. Logistic regression showed that an admission Glasgow Coma Scale (GCS) score of less than 8 points (odds ratio = 4.261, 95% confidence interval 1.102–16.476), hyperglycaemia (2.759, 1.159–6.564), hypothermia treatment (6.557, 2.244–19.159), and central venous catheterisation (CVC) (8.853, 2.860–27.398) were independent risk factors for HAIs in patients with intracranial aneurysm who underwent surgery.

**Conclusion:**

Being comatose upon hospital admission, having hyperglycaemia or hypothermia, and indwelling CVC are major risk factors for HAIs in patients undergoing surgery for intracranial aneurysms.

## Introduction

An intracranial aneurysm is a bulged, weakened area in the wall of an artery in the brain. Surgical intervention before aneurysm rupture is the main method used to reduce the risk of bleeding and mortality, however surgical and surgery-related factors increase the risk of HAI [1]. Patients with ruptured intracranial aneurysms often have severe illness, spend a long time hospitalized and being bedridden, and experience complications such as trouble of consciousness and other neurological impairments. After craniotomy, patients are more prone to HAI as a consequence of the length and difficulty of this operation. HAI can aggravate the patient’s condition, seriously interfere with clinical treatment, prolong the patient’s hospitalization time, increase their treatment costs and re-admission rate within 30 days, and lead to severe disability and death. Thus, it causes medical disturbances and increases the economic burden on society and individuals [2, 3].

Prevention and control of infection is the cornerstone of patient safety procedures, and HAI is a serious threat to patient safety [4]. A large number of studies have confirmed that interventions using evidence-based strategies can prevent the occurrence of HAI [5]. Accurate identification of the risk factors associated with HAI and early prevention and control play important parts in reducing its incidence. In recent years, investigations and analyses have been conducted in various fields, from various perspectives, to determine the factors affecting HAI and to develop interventions. Amey et al [6] analysed the risk factors for respiratory infection in 103 patients with aneurysmal subarachnoid haemorrhage clipping. Postoperative tracheal intubation of more than 48 h, tracheotomy, and stay in the intensive care unit (ICU) of more than 5 days were independent risk factors for respiratory infection. Frontera et al. [7] and Cinotti et al. [8], and others found that the risk factors for HAI in patients with subarachnoid haemorrhage included age, sex, poor clinical condition upon hospital admission, ICU admission, oral intubation, tracheotomy, and urethral/ventricular drainage. Various risk factors for infection have been identified in different research populations. A few studies on HAI in patients with intracranial aneurysms have been conducted. The epidemiological and risk factors for nosocomial infection in patients with intracranial aneurysms need to be further explored.

In addition, previous studies on risk factors and interventions for HAI have mainly focused on the role of doctors; only few studies have considered the perspective of nurses. Nursing staff represent the clinical front line in terms of staff contact with patients, and they have an indispensable part to play in hospital infection control [9–11]. For that reason, this study considers HAI from the perspective of the nursing staff. The research objectives are as follows: to describe the epidemiology of HAI in patients with intracranial aneurysms; to determine the risk factors associated with HAI in patients with intracranial aneurysms; and to analyse the relationship between HAI and patient prognosis.

## Methods

### Subjects

Patients who were admitted to the Department of Neurosurgery, Xuanwu Hospital of Capital Medical University, between the 1st of January and the 31st of December 2016, were selected according to the following criteria. Inclusion criteria: patients diagnosed with intracranial aneurysms by neurosurgical physicians combined with clinical signs and symptoms, imaging data, and laboratory data; patients undergoing surgery and intervention during hospitalization. Exclusion criteria: patients who had been diagnosed or were in latent infection before admission. Elimination criteria: patients who were missing more than 10% of their medical records. Participants were divided into an infection group (*n* = 64) and a control group (*n* = 478). The control group had patients without hospital-acquired infections. This study was approved by the Xuanwu Hospital Ethics Review Committee (Clinical Research Council [2017]024).

### Method

This was a retrospective case-control study. According to the selection criteria, the retrospective medical information survey method [12, 13] was used to collect patient medical data using a self-designed form, which included the patient’s basic information, diagnosis and treatment data, disease-related symptoms and signs, as well as test and imaging results. Basic data (age, gender, diagnosis, history, etc.), laboratory data (leucocyte, albumin, blood glucose, liver function, renal function, electrolytes, etc.), and treatment information (length of stay in ICU, mechanical ventilation, surgery, postoperative blood transfusion, hypothermia treatment, central venous catheterization, etc.) were collected. Outcome at discharge was assessed by the Glasgow Outcome Score (GOS).

### Diagnostic criteria

The diagnostic criteria were based on the *Diagnostic Standards for Hospital Acquired Infections (Trial)* issued by the Ministry of Health of the People’s Republic of China on 3 January 2001 to define the criteria for diagnosis of HAI [14]. This study included diagnoses of hospital infections involving the respiratory system, blood system, central nervous system, and urinary system.

### Statistical analysis

EpiData 3.0 was used for data entry and system logic error detection. Statistical analysis was performed using SPSS 22.0. The count data is presented as frequency (percentage) and the differences between groups were assessed using the χ^2^ test. The normally distributed continuous data is presented as mean ± standard deviation, and the non-normally distributed continuous data is presented as median (P25, P75). When the data was normally distributed and the between-group variance was equal, differences between groups were assessed using *t*-tests; the rank sum test was used when the data showed a biased distribution. Univariate analysis was used to perform preliminary variable selection, using an α level of 0.1. On the other hand, logistic stepwise regression analysis was used to analyse the independent predictors of HAI in patients with intracranial aneurysms; differences with *P* < 0.05 were considered statistically significant.

## Results

### Occurrence of HAI

A total of 542 patients with neurosurgical intracranial aneurysms were enrolled, including 225 males (41.5%) and 317 females (58.5%), aged 18–83 years, with an average age of 54.5 ± 11.8 years. A total of 77 cases of hospital infections occurred in 64 patients, with an infection rate of 11.8% and an infection case rate of 14.2%. The most common type was respiratory infections, with 49 cases (63.6%), followed by infections involving the blood system (16 cases, 20.8%), central nervous system (10 cases, 13.0%), and urinary system (only two cases, 2.6%) (Table 1). Hospital infections occurred 1 to 18 days after surgery, with an average occurrence of 5 days after surgery.

**Table 1:** HAI site and composition ratio

Infection site	Infection cases	Composition ratio (%)
Respiratory system	49	63.6
Blood system	16	20.8
Central nervous system	10	13.0
Urinary system	2	2.6
Total	77	100.0

### Univariate analysis of HAI

Univariate analysis indicated that lower activities of daily living score on admission, emergency admission, mechanical ventilation, taking antiplatelet aggregation drugs, admission GCS score of less than 8 points, high Hunt–Hess grade on admission, albumin reduction, hyperglycaemia, hyponatremia, surgical procedure, unexpected reoperation, operation time > 4 h, non-class I incision, intraoperative blood loss, hypothermia therapy, and central venous catheterization were factors associated with HAI in patients with intracranial aneurysm surgery (*P* < 0.05) (Table 2).

**Table 2:** Univariate analysis of HAI

Relevant factors	Infection group (*n* = 64)	Control group (*n* = 478)	t/z/χ^2^	*P*
Gender n (%)				
Male	31 (48.4)	194 (40.6)	1.433	0.231
Female	33 (51.6)	284 (59.4)		
Age n (%)				
≤ 44 years old	8 (12.5)	79 (16.5)	2.737	0.255
45–59 years old	28 (43.8)	239 (50)		
≥ 60 years old	28 (43.8)	160 (33.5)		
Admission Activities of daily living (ADL) M(Q)	30 (0–64)	95 (45–100)	−6.699	0.000
Admission route n (%)				
Emergency	47 (73.4)	191 (40)	25.687	0.000
Elective	17 (26.6)	287 (60)		
Hospitalization time M(Q)	19 (15–24)	8 (6–12)	−10.295	0.000
Mechanical ventilation n (%)				
Yes	48 (75)	76(15.9)	111.735	0.000
No	16 (25)	402(84.1)		
Hypertension n (%)				
Yes	23 (35.9)	169 (35.4)	0.008	0.927
No	41 (64.1)	309 (64.6)		
Diabetes n (%)				
Yes	9 (14.1)	69 (14.4)	0.006	0.936
No	55 (85.9)	409 (85.6)		
Coronary heart disease n (%)				
Yes	5 (7.8)	19 (4)	1.964	0.161
No	59 (92.2)	459 (96)		
Immune system disease n (%)				
Yes	1 (1.6)	7 (1.5)	0.004	0.952
No	63 (98.4)	471 (98.5)		
Steroids n (%)				
Yes	12 (18.8)	115 (24.1)	0.887	0.346
No	52 (81.3)	363 (75.9)		
Antiplatelet aggregate n (%)				
Yes	11 (17.2)	221 (46.2)	19.452	0.000
No	53 (82.8)	257 (53.8)		
Admission GCS score n (%)				
≤ 8 points	25 (39.1)	19 (4)	93.161	0.000
> 8 points	39 (60.9)	459 (96)		
Admission Hunt-Hess classification n (%)				
≤ 2 points	35 (54.7)	435 (91)	64.623	0.000
≥ 3 points	29 (45.3)	43 (9)		
Leukopenia n (%)				
Yes	2 (3.1)	19 (4)	0.109	0.741
No	62 (96.9)	459 (96)		
Albumin reduction n (%)				
Yes	14 (21.9)	36 (7.5)	13.867	0.000
No	50 (78.1)	442 (92.5)		
Hyperglycaemia n (%)				
Yes	45 (70.3)	166 (34.7)	30.062	0.000
No	19 (29.7)	312 (65.3)		
Abnormal liver function n (%)				
Yes	6 (9.4)	43 (9)	0.010	0.921
No	58 (90.6)	435 (91)		
Abnormal renal function n (%)				
Yes	3 (4.7)	5 (1)	2.947	0.086
No	61 (95.3)	473 (99)		
Hyperkalaemia n (%)				
Yes	1 (1.6)	1 (0.2)	0.335	0.562
No	63 (98.4)	477 (98.8)		
Hypokalaemia n (%)				
Yes	9 (14.1)	54 (11.3)	0.420	0.517
No	55 (85.9)	424 (88.7)		
Hypernatremia n (%)				
Yes	3 (4.7)	14 (2.9)	0.142	0.707
No	61 (95.3)	464 (97.1)		
Hyponatremia n (%)				
Yes	12 (18.8)	26 (5.4)	13.365	0.000
No	52 (81.3)	452 (94.6)		
Surgical approach n (%)				
Intervention	22 (34.4)	320 (66.9)	25.716	0.000
Craniotomy	42 (65.6)	158 (33.1)		
Surgery duration n (%)				
≤ 4 h	30 (46.9)	365 (76.4)	24.825	0.000
> 4 h	34 (53.1)	113 (23.6)		
Non-class I incision n (%)				
Yes	1 (1.6)	2 (0.4)	0.964	0.326
No	63 (98.4)	476 (99.6)		
Surgical level n (%)				
Small	0	1 (0.2)	6.914	0.075
Medium	1 (1.6)	32 (6.7)		
Large	4 (6.3)	9 (1.9)		
Extra large	59 (92.2)	436 (91.2)		
Anaesthesia method n (%)				
General anaesthesia	64 (100)	465 (97.3)	0.811	0.368
Local anaesthesia	0	13 (2.7)		
Intraoperative blood loss n (%)				
< 1000 ml	60 (93.8)	473 (99)	6.445	0.011
≥ 1000 ml	4 (6.3)	5 (1)		
Unexpected reoperation n (%)				
Yes	8 (12.5)	8 (1.7)	19.468	0.000
No	56 (87.5)	470 (98.3)		
Hypothermia treatment n (%)				
Yes	26 (40.6)	11 (2.3)	124.376	0.000
No	38 (59.4)	467 (97.7)		
Artificial airway n (%)				
Yes	64 (100)	466 (97.5)	0.688	0.407
No	0	12 (2.5)		
Indwelling catheter n (%)				
Yes	64 (100)	474 (99.2)	1.009	0.315
No	0	4 (0.8)		
Central venous catheter n (%)				
Yes	58 (90.6)	119 (24.9)	110.883	0.000
No	6 (9.4)	359 (75.1)		

### Multivariate analysis of HAI

Multivariate analysis was performed for the variables for which *P* < 0.1 in the univariate analysis. From the logistic regression results, it appeared that admission GCS score ≤ 8 points (odds ratio = 4.261, 95% confidence interval 1.102–16.476), hyperglycaemia (2.759, 1.159–6.564), hypothermia treatment (6.557, 2.244–19.159), and CVC (8.853, 2.860–27.398) were independent risk factors for HAI in patients with intracranial aneurysm surgery (Table 3).

**Table 3:** Multivariate analysis of HAI

Variable	*B*	*S.E.*	*Wald*	*P*	*OR*	*95% CI*
Admission GCS score ≤ 8 points	1.450	0.690	4.414	0.036	4.261	1.102–16.476
Hyperglycaemia	1.015	0.442	5.264	0.022	2.759	1.159–6.564
Hypothermia treatment	1.881	0.547	11.816	0.001	6.557	2.244–19.159
Central venous catheter	2.181	0.576	14.313	0.000	8.853	2.860–27.398
Constant	−4.001	1.753	5.209	0.022	0.018	–

### Clinical outcomes

This study included 542 cases of intracranial aneurysms and eight deaths in 2016; the mortality in the infection group was 4.7% and that of the control group was 1%. The proportions with GOS score ≤ 3 in the infection group and the control group were 70.3 and 9.6%, respectively. The average hospitalization durations in the infection group and the control group were 19 days and 8 days respectively, and the difference was statistically significant (*P* = 0.000). The average hospitalization cost for the 64 infected patients was 205,853 Renminbi (RMB), while that for patients without hospital infection was 107,505 RMB. The medical expenses of the infection group were 1.9 times those of the control group. On average, each infected case cost 98,348 RMB more than an uninfected one. The difference was statistically significant (*P* = 0.000). The average antibacterial drug fee for patients in the infection group was 1059 RMB, compared with 280 RMB for patients without hospital infection; the difference was statistically significant (Table 4).

**Table 4:** Clinical outcomes

	Infection group (*n* = 64)	Control group (*n* = 478)	Statistics	*P*
GOS score n (%)				
Mortality; GOS = 1	3 (4.7)	5 (1.0)	110.413	0.000
Poor outcome; GOS = 2/3	42 (65.6)	41 (8.6)		
Good outcome; GOS = 4/5	19 (29.7)	432 (90.4)		
Hospitalization time (day)	19 (15–24)	8 (6–12)	−10.295	0.000
Hospitalization cost (RMB)	205,853 (142,293–289,816)	107,505 (76,548–153,882)	−7.381	0.000
Antibacterial drug fee (RMB)	10,059 (5,021–19,570)	280 (0–398)	−12.243	0.000

## Discussion

### Current status of HAI

HAI is a difficult issue and a key point in the clinical diagnosis and treatment of neurosurgery, especially in patients with severe neurosis; it is also an important factor affecting clinical treatment and patient prognosis [12]. Previous studies found that the incidence of HAI in non-Chinese neurosurgical hospitals was 6.7–11.1% [15, 16], while the incidence in Chinese hospitals is 5.74–11.32% [17–20]. In this study, the infection rate of patients with intracranial aneurysms was 11.8%, and the infection case rate was 14.2%. When compared with the overall infection rate of neurosurgery, the high infection rate may be related to the characteristics of the patients. Of the patients surveyed, 267 (49.3%) were admitted to the ICU, 124 (22.9%) were mechanically ventilated, and 530 (97.8%) had indwelling artificial airways. Respiratory infections in hospital infections ranked first in this study (63.6%), higher than the 2014 national hospital infection cross-sectional survey report [21] in which the incidence of respiratory infection was 47.53%. The patients affected had impaired neurological function, multiple disturbances of consciousness, limited self-care ability, long periods of being bedridden, difficulty with sputum drainage, and invasive operation of the respiratory tract. In view of the preceding, prevention and treatment of respiratory infections should be the focus in neurosurgical hospitals, including shortening the time of surgical anaesthesia intubation and postoperative indwelling intubation, and focusing on turning over and draining. In this study, hospital infections occurred 1 to 18 days after surgery, with an average of 5 days after surgery. This is similar to the report by Laban [22] in which hospital infection after subarachnoid haemorrhage occurred in the first week after acute onset. It is suggested that the week following the operation is a high-risk period for hospital infection. This period should thus be considered crucial for the prevention and control of hospital infection.

### Risk factors for HAI

Hospital infections in postoperative patients with intracranial aneurysms can be affected by a variety of factors. This study shows that patients admitted to hospital with GCS ≤ 8 points, hyperglycaemia, hypothermia, and indwelling central venous catheterization are independent risk factors for nosocomial infection.

Patients with coma manifest disturbances of consciousness with inhibition of normal physiological reflexes such as swallowing and coughing, resulting in increased respiratory tract secretions and susceptibility to bacterial reproduction. In addition, patients with coma tend to suffer from severe diseases, with the need for tracheal intubation, tracheotomy, mechanical ventilation, nasogastric stomach tube, or urinary tract intubation-assisted treatment. Such operations can damage the mucosa and decrease its barrier function; this is an important risk factor for nosocomial infection [13, 23]. Therefore, patients with coma should be actively treated and their early waking encouraged, as well as strictly controlling the indications for invasive operations, performing operations under strict aseptic conditions, promoting gentle movement, keeping the airway open, and ensuring the removal of deep sputum to reduce the incidence of infection.

Consistent with previous studies, the results indicate that hyperglycaemia is another risk factor for nosocomial HAI [24]. Hyperglycaemia increases plasma osmotic pressure of patients, inhibits the chemotaxis activity, adhesion ability, phagocytic ability and intracellular killing effect of leucocyte, and reduces the body’s resistance to infection. In addition, long-term hyperglycaemia favours bacterial reproduction, especially in the respiratory tract, urinary tract, skin, and female patients’ genital area, which increases the risk of infection [25]. Consequently, attention should be paid to the monitoring and management of blood sugar and control of glucose intake, as well as whether diabetic patients develop a further increase in blood sugar after trauma, or non-diabetic patients experience stress hyperglycaemia as a result of trauma, surgery, or other stress conditions [26].

Of the patients undergoing aneurysm surgery and admitted to our department in 2016, 37 patients were treated with hypothermia. Their targeted temperature range was 33–35 °C and the continuous treatment time range was 1–10 days. 26 out of 37 (70.3%) had hospital infections. This incidence rate was significantly higher than that among non-hypothermia patients. In the infection group, 24 (64.9%) of infection cases were respiratory infections and six (16.2%) were bloodstream infections. Shiozaki et al [27] found that the incidence of respiratory infection in patients treated with hypothermia was 49%, lower than that found in this study. Hypothermia treatment is a common therapeutic schedule for patients undergoing neurosurgery, especially those with severe craniocerebral injury, as it can reduce brain metabolism and thus limit brain damage. This is particularly important for patients with severe cerebral vasospasm and severe intracranial pressure, as it can reduce the gap between effective cerebral perfusion and the cerebral metabolic needs of brain blood flow [28]. On the other hand, hypothermia may lead to decreased systemic immune function. Patients treated with hypothermia treatment were accompanied by mechanical ventilation and sedative analgesic medication. The use of mechanical ventilation may lead to deep airway bacterial colonization in patients, and sedative analgesic medication may cause cough reflexes to be inhibited [29]. These may aggravate and/or lead to infection and other complications [30]. It has been suggested that for patients receiving hypothermia treatment, nursing staff should not only cooperate with doctors to carry out effective body temperature control for brain protection, but also pay attention to infection control, pipeline care, skin care, environmental monitoring, and respiratory management.

Patients undergoing neurosurgery tend to experience severe surgical trauma and rapid changes in condition; they are also bedridden for long periods. Due to the need for monitoring and treatment, central venous catheterization is widely used in clinical practice. In this study, 177 patients (32.7%) had indwelling central venous catheters. Although central venous catheterization is a relatively mature and routine technique [31], such invasive procedures damage the tissue mucosa, destroying the body’s normal defence barrier. This allows certain conditional pathogens to invade the patient’s body, or ectopic implantation of the normal flora to take place. The probability of infection increases with the extension of catheter retention time [9]. The results of this study were consistent with previous reports in this regard; 58 patients (32.8%) with catheterization had hospital infections, significantly more than the six (1.6%) patients without catheterization. Therefore, daily assessment of the need for an indwelling central venous catheter and early removal of the catheter are significant tasks when it comes to prevention and control of hospital infections. During the indwelling of the central venous catheter, the nursing staff should perform maintenance of the catheter by strictly replacing the infusion and static push drugs; replacement of the catheter dressing is a delicate mandate and should be done by trained intravenous infusion nurses to prevent the occurrence of hospital infections.

This was a single-centre, retrospective study, although its results were confirmed by a multicentre prospective study. Furthermore, this study did not provide long-term follow-up of patients. The long-term impact of HAI on patients with intracranial aneurysms and their prognosis is not yet clear and needs to be further studied.

## Conclusions

In summary, the incidence of hospital infections is higher in patients with intracranial aneurysms, it is especially the case of respiratory infections. Coma on admission, hyperglycaemia, hypothermia, and indwelling central venous catheterization are major risk factors for HAI in patients with intracranial aneurysms. Hospital infections affect a patient’s prognosis, prolongs their hospitalization time, and increase their medical expenses. The amount of hospital infections could be reduced by training medical staff on infection and prevention, strengthening the monitoring and management of susceptibility factors and risk factors for high-risk patients with neurosurgical hospital infections, and actively taking corresponding preventive measures. Standardized diagnosis and treatment of patients with hospital infections, through multidisciplinary collaboration and the cooperation and support of relevant professionals, would promote early control of hospital infections and improve patients’ prognosis and outcomes.

## Data Availability

The datasets obtained during this study are available from the corresponding author on reasonable request.

## Abbreviations

ADL: Activities of daily living
CVC: Central venous catheterisation
GCS: Glasgow Coma Scale
GOS: Glasgow Outcome Scale
HAI: Hospital-acquired infection
ICU: Intensive care unit
RMB: Renminbi
